# Is the continuum of coercion in psychiatry really a continuum? A statistical implication analysis

**DOI:** 10.1080/13218719.2024.2377580

**Published:** 2024-09-08

**Authors:** Philippe Golay, Benedetta Silva, Debora Martinez, Mizué Bachelard, Nolan Pedro Fernando, Jonathan Perrin, Charles Bonsack, Stéphane Morandi

**Affiliations:** a Service of Community Psychiatry, Department of Psychiatry, Lausanne University Hospital and University of Lausanne, Switzerland; b Service of General Psychiatry, Department of Psychiatry, Lausanne University Hospital and University of Lausanne, Switzerland; c Cantonal Medical Office, Directorate General for Health of the Canton of Vaud, Department of Health and Social Action, Lausanne, Switzerland

**Keywords:** informal coercion, perceived coercion, Rasch analysis, statistical implication, treatment pressures

## Abstract

Formal coercion in psychiatry is widely studied, but less is known about informal coercion. Coercion is mostly viewed as a continuum, with formal coercion considered the most severe. The question of whether informal coercion might be perceived as even more severe remains unevaluated. We aimed to compare the perceived severity of formal coercion among psychiatric patients with four domains of severe informal coercion (finance, housing, criminal justice and child custody). A total of 456 psychiatric patients were evaluated using Rasch analysis and statistical implication analysis. All four domains of severe informal coercion were found to be more severe than formal coercion, with coercion involving child custody perceived as the most severe. The perceived seriousness of severe informal coercion may be a counter-example to the idea of a ‘continuum of coercion’. The degree of severity of formal and informal coercion may not correspond to the lived experiences of the patients concerned.

## Introduction

Coercion in psychiatry is justified by the need to protect either patients or third parties. Formal coercion consists of the legal procedures used to force someone into treatment. Informal coercion comprises various forms of pressure that medical staff or relatives can use to persuade someone to undergo treatment (Szmukler & Appelbaum, 2008).

Coercion’s negative impact has been discussed numerous times (Katsakou et al., 2011; Kinner et al., 2017; Nyttingnes et al., 2016; Rüsch et al., 2014; Theodoridou et al., 2012), with formal coercion shown to negatively impact patients’ quality of life (Rüsch et al., 2014) and their clinical course. These lead to decreased satisfaction with care (Nyttingnes et al., 2016) and worse treatment adherence in the long term (de Haan et al., 2007).

Much less is known about treatment pressure and informal coercion and their potential adverse effects (Hotzy & Jaeger, 2016). Lovell (1996) described four forms of social control over psychiatric patients and represented them on a continuum from the most to the least coercive: coercion, coerced voluntarism, utilitarian compliance and persuasion. Lidz et al. (1998) proposed a distinction between positive pressures and negative pressures within informal coercion. The key difference between these symbolic pressures lies in the willingness to encourage or threaten the person. Angell (2006) also developed a continuum of six forms of coercive strategies used by practitioners to maintain treatment compliance: persuasion, monitoring, incentives, leverage, threats and using the authorities. The most widespread model of informal coercion in the literature is probably Szmukler and Appelbaum’s (2008) five-category description: persuasion, interpersonal leverage, inducements, threats and compulsory treatment. Persuasion is the act of appealing to the patient’s reason and emotions to persuade them to accept a therapeutic measure. Interpersonal leverage is the act of using the emotional bond that professionals or relatives have with the patient to convince them to agree to a therapeutic intervention. Inducement is the act of making certain benefits (e.g. cigarettes, money, shelter) conditional on the patient accepting a therapeutic intervention (e.g. taking their medication). A threat is the act of suggesting that the patient will lose something (e.g. money, housing benefits, their freedom) if they refuse a therapeutic intervention. Finally, compulsion or formal coercion is the act of legally forcing someone to accept inpatient or outpatient treatment. Drawing on Szmukler and Appelbaum (2008), Klingemann et al. (2022) recently suggested a new, three-category model of informal coercion: treatment pressure, informal coercion and formal coercion. Treatment pressure is defined as a mild form of coercion whose use does not prevent the patient from making autonomous choices. On the contrary, the informal coercion category is described as a more restrictive practice in which caregivers or relatives use threats, someone else’s decisions and force to oblige the patient to undergo treatment.

As mentioned above, informal and formal coercion are usually described as a continuum, with the use of formal coercion considered the most coercive action. However, the question of whether informal coercion could, in some instances, be perceived as more severe than formal coercion remains largely open. Severity could be defined in many ways, using the lived experiences and perceptions of the people involved or using a moral standpoint. Severity could also be classified based on a coercive measure’s pattern of use. Severity could be considered from the perspective of implication: the use of a specific severe form of coercion could imply that other forms of coercion had also been used beforehand. On the other hand, the use of a coercive measure judged as less severe would not imply that other measures had not been used.

The goal of the present study, therefore, was to explore and compare the use of formal coercion to several domains of severe informal coercion involving finance, housing, criminal justice and child custody. Severity was defined by the pattern of use of coercive measures. The overarching objective was to estimate the severity of each form of coercion with psychiatric patients by combining a classic Rasch model approach with the novel statistical approach of directly studying the respective implicative relationships between different forms of coercion.

## Material and methods

### Participants

Participants were recruited between March 2020 and April 2023 via six psychiatric hospitals in the French-speaking regions of Switzerland and an online survey. The inclusion criterion was that participants should be at least 18 years old and no older than 65. People diagnosed with dementia (F00–F09) or an intellectual disability (F70–F79) were excluded.

Potential participants in the hospitals were contacted directly by trained research assistants and asked to take part in the study voluntarily. The online survey was advertised on various social media platforms and relayed by patients’ associations. Potential online survey participants were informed that they could take part in the study if they were or had been in psychiatric care, had a diagnosed psychiatric disorder and had a sufficient level of French. A correct answer to the control item (‘*In order to check your concentration, please answer* *“Rather* *yes”* *to this question’*), to be aged between 18 and 65 years, and to have completed sociodemographic and diagnostic data were required in order be included in the analysis.

The Human Research Ethics Committee of the Canton of Vaud, Switzerland, approved the study (protocol #2016–00768). Informed consent was obtained from all participants, and all our research was carried out in accordance with the recommendations of the Human Research Ethics Committee of the Canton of Vaud and the Declaration of Helsinki.

### Measures

Patients were asked to report their gender, age and most significant ICD-10 diagnosis. In some instances, patients were assessed during their first psychiatric hospitalisation, and no diagnostic information was yet available.

#### Informal coercion

Pressure to adhere to treatment (‘leverage’) was assessed using a four-item instrument proposed by Burns et al. (2011) and adapted from Monahan et al. (2005). This instrument aims to measure patients’ history or experiences of being subjected to leverage in four domains of social welfare: finance, housing, the criminal justice system and child custody (Table 1). These items represent rather severe forms of informal coercion. They correspond to the domains of inducement and threat defined by Szmukler and Appelbaum (2008). Participants’ answers were dichotomous (yes/no). Despite the relative crudeness of its rating system, this instrument has been shown to be significantly correlated with another more continuous and well-validated measure of perceived pressures in psychiatry (Golay et al., 2024). It also has the benefit of distinguishing four distinct domains within which coercion can be exerted, which is not possible with a single overall score.

**Table 1.** Informal coercion items.

Domains	Item description
Finance	Did anyone (financial manager/guardian) ever make giving you your money, or giving you spending money, depend on whether you did what he or she wanted in terms of getting mental health, alcohol or drug treatment (or taking medication)?
Housing	Have you ever lived somewhere where you were required to stay in mental health or substance use treatment (or required to continue taking your medication) to keep living there (including family home)? Have you ever been told that obtaining new accommodation is dependent on you taking treatment?
Criminal justice	Has anyone in the legal system ever told you or your lawyer that the charges will be dropped or reduced if you get treatment in the community for your mental health, alcohol or drug problems? Has a mental health professional, or anyone in the legal system, or their report, suggested that you take treatment for a mental health problem as a condition of not going to prison?
Child custody	Have you ever been told that your children might be taken into care if you did not participate in mental health, alcohol or drug treatment (or taking your medication)? Have you ever been told that your access to see your children would be reduced if you did not participate in mental health, alcohol or drug treatment (or taking your medication)?

Note. from Burns et al., 2011.

#### Formal coercion

Having experienced formal coercion was defined as having experienced compulsory formal coercive measures such as involuntary hospitalisation, confinement to an institution, restrictions on freedom of movement, seclusion, forced medication and physical restraint. In Switzerland, before any formal coercive measures can be implemented, such as forced medication, seclusion or restraint, involuntary hospitalisation is mandatory. Patients were provided with this definition and asked to report whether they had ever undergone formal coercion (yes/no). Therefore, formal coercion involves involuntary hospitalisation, at the very least.

### Statistical analysis

To estimate the severity of different forms of informal and formal coercion (involving finance, housing, criminal justice, child custody and formal coercion), we first used a Rasch model. Originally part of Item Response Theory (IRT), this model describes the relationship between the probability of the occurrence of one form of coercion and the level of a patient on a latent continuum. This latent continuum represents the amount of coercion experienced. This one-parameter model describes the presence of probabilities using a series of logit regression lines with identical slopes (De Ayala, 2013). The locations of the slopes along the continuum correspond to the items’ severity parameters. This severity parameter represents the level of coercion experienced needed to expect a 50% probability of the presence of another given measure. The Rasch model, therefore, defines severity as a point along a continuum, and it assumes that, for any given level of coercion experienced, a more severe coercive measure will be less frequently reported than a less severe coercive measure. The Rasch model was estimated using a robust weighted least squares estimator with adjustments for the mean and variance (WLSMV). Several indicators of model fit were used: the Root Mean Square Error of Approximation (RMSEA), the Comparison Fit Index (CFI) and the Tucker–Lewis fit Index (TLI). RMSEA values ≤ 0.06, and CFI and TLI values ≥ 0.95, were interpreted as good fits, whereas RMSEA values ≤ 0.08, and CFI and TLI values ≥ 0.90, were considered indicative of a satisfactory fit (Hu & Bentler, 1998).

Next, we estimated whether some forms of coercion implied that other forms of coercion had already been attempted. Oriented dependencies of the different forms of coercion were estimated using the Iota statistical implication index for dichotomous variables (Noël, 2021). Unlike symmetrical indices, such as correlation coefficients, asymmetrical bidirectional relationships can also be distinguished. An example of an asymmetrical relationship might be a child’s ability to walk in relation to tying their shoes. Knowing how to tie one’s shoes will almost always imply knowing how to walk, whereas knowing how to walk will not systematically imply knowing how to tie one’s shoes. ιA⇒B allows us to estimate whether the presence of A implies that B will also be present, whereas ιB⇒A allows us to estimate whether the presence of B implies that A will also be present. Naturally, both relationships can take different values. ιA⇒B varies from –∞ to +∞, with positive values showing evidence in favour of A implying B, 0 representing uncertainty and negative values showing evidence against this implication (Noël, 2021). These coefficients can be tested for statistical significance and compared, with a higher coefficient indicating a stronger implication. The Iota implication index was chosen because it is superior to other coefficients used to estimate the strength of implication based solely on the rarity of counter-examples (Noël, 2021). In contrast, the Iota coefficient is also able to exploit counter-positive information (e.g. when ‘A implies B’, the counter positive is ‘not B implies not A’). The Iota coefficient’s statistical significance was fixed at .05 and was determined using a bootstrap method. The index’s 95% confidence interval was computed and checked for whether it contained zero. All statistical analyses were performed using IBM SPSS 27 software, the Mplus statistical package (version 8.3) and the ‘boot’ package for R software (Canty & Ripley, 2016).

## Results

A total of 456 patients participated; 229 (50.2%) were women. Ages ranged from 18 to 64 years (*M* = 39.04, *SD* = 13.25). Primary diagnoses, based on the *International Statistical Classification of Diseases and Related Health Problems 10th Revision* (ICD-10, World Health Organization, 1993), are presented in Table 2. A history of formal coercion was reported by 267 (58.6%) participants, with a history of severe informal coercion reported less frequently, ranging from 23.0% for coercion involving housing to 6.8% for coercion involving child custody. Severe informal coercion involving finance and the criminal justice system were both reported by 10.3% of the participants.

**Table 2.** Sample description.

Variable	*M* (*SD*)	% (*n*)
Age	39.04 (13.25)	
Gender		
Female		50.2 (229)
Male		47.1 (215)
Other		2.2 (10)
Prefer not to say		0.4 (2)
Recruited		
In hospital		85.7 (391)
Online		14.3 (65)
Lifestyle		
Private household, living alone		44.0 (200)
Private household, living with several people		45.1 (205)
Sheltered institution/accommodation		7.5 (34)
Homeless		2.2 (10)
Other		1.3 (6)
Marital status		
Single		61.4 (280)
Married		16.2 (74)
Registered partnership		0.7 (3)
Divorced		14.5 (66)
Separated		5.5 (25)
Widow		1.8 (8)
Education		
Education not completed		0.9 (4)
Compulsory education		12.1 (55)
Apprenticeship		24.6 (112)
Baccalaureate/high school diploma		20.8 (95)
Professional/commercial/technical school		12.9 (59)
University		17.8 (81)
Other		11.0 (50)
Most significant diagnosis (ICD-10)		
Mental and behavioural disorders due to alcohol use (F10)		5.9 (27)
Mental and behavioural disorders due to psychoactive substance use (F11–F19)		3.9 (18)
Schizophrenia (F20–F29)		23.5 (107)
Mood affective disorders – mania (F30–F31)		12.3 (56)
Mood affective disorders – depression (F32–F39)		28.3 (129)
Neurotic, stress-related and somatoform disorders (F40–F48)		7.5 (34)
Personality disorders (F60–F69)		13.4 (61)
Psychological development disorders (F80–F89)		0.2 (1)
No diagnostic information available (first psychiatric hospitalisation)		3.9 (18)
History of informal coercion involving finance		10.3 (47)
History of informal coercion involving housing		23.0 (105)
History of informal coercion involving criminal justice		10.3 (47)
History of informal coercion involving child custody		6.8 (31)
History of formal coercion		58.6 (267)

Note. *N* = 456.

The goodness of fit indices indicated that the Rasch model had a satisfactory fit to the data (RMSEA = 0.052, CFI = 0.936, TLI = 0.929). Severity parameters (Table 3) indicated that a history of formal coercion could be considered the least severe item, followed by a history of informal coercion involving housing. Informal coercion involving finance and the criminal justice system had the same severity parameter. Informal coercion involving child custody was perceived to be the most severe form of coercion.

**Table 3.** Rasch model items severity parameters.

Items	Severity parameter
Formal coercion	−0.336
I.C. Housing	1.149
I.C. Finance	1.968
I.C. Criminal justice	1.968
I.C. Child custody	2.321

Note. Severity parameters in ascending order. I.C. = Informal coercion.

The results of our statistical implication analyses revealed that a history of informal coercion involving child custody implied all other forms of informal coercion (Table 4). Informal coercion involving housing, in contrast, hardly implied any other form of informal coercion. Finance and criminal justice fell in the middle and implied each other symmetrically. When formal coercion was taken into account, all four domains of severe informal coercion strongly implied a history of formal coercion. In the other direction, negative coefficients revealed evidence against formal coercion implying any of the four domains of informal coercion. In other words, formal coercion did not statistically imply a severe form of informal coercion.

**Table 4.** Oriented dependencies between the different forms of coercion.

Variable A	Variable B	Iota statistical implication index (Noel, 2021)	
		ιA⇒B	ιB⇒A
I.C. Finance	I.C. Housing	2.833*	0.251
I.C. Finance	I.C. Criminal justice	1.152*	1.152*
I.C. Finance	I.C. Child custody	0.082	1.041
I.C. Finance	Formal coercion	3.970*	−2.120*
I.C. Housing	I.C. Criminal justice	0.577	3.546*
I.C. Housing	I.C. Child custody	−0.775*	2.401*
I.C. Housing	Formal coercion	3.248*	−0.922*
I.C. Criminal justice	I.C. Child custody	1.042*	2.175*
I.C. Criminal justice	Formal coercion	6.209*	−1.842*
I.C. Child custody	Formal coercion	4.845*	−2.550*

Note. I.C. = Informal coercion.

*p < .05.

## Discussion

Overall, Rasch analysis and statistical implication analysis both suggested that the use of informal coercion in its strongest forms was more severe than formal coercion. Experiencing severe informal coercion statistically implied more experiences of formal coercion, whereas experiencing formal coercion did not imply severe informal coercion, and this held for all four domains of informal coercion assessed. This may constitute a counter-example to the idea of the ‘continuum of coercion’, which implicitly states that formal coercion is the most severe form.

Within the four domains of informal coercion, coercion involving custody of the patient’s children was perceived as the most severe and coercion involving housing was perceived as the least severe. Perceptions of coercion involving the criminal justice system and finances were similar. It should be kept in mind that not all patients have children, but they are likely all concerned about their housing, the criminal justice system or their finances. Other types of coercion could, therefore, less easily imply pressure occurred in the domain of child custody. Nevertheless, statistical implication in the opposite direction still suggested that, when present, leverage involving child custody strongly implied a history of all the other forms of coercion.

These results confirmed the paramount importance of not limiting studies on perceived coercion to patients who have experienced formal coercive measures. Indeed, several previous studies have pointed out the limitations of this approach (Bonsack & Borgeat, 2005; Golay et al., 2019; Iversen et al., 2002; Monahan et al., 1995; O’Donoghue et al., 2014) and highlighted the need to investigate a broader range of coercive experiences (Mårtensson & Fridlund, 2017; Russo & Wallcraft, 2011). However, very few studies have considered the lived experiences of coercion of voluntary inpatients (Silva et al., 2023).

To broaden the study of coercion to include informal coercion, better measurement tools need to be developed. To date, only the four-item instrument used in this study covers a wide variety of contexts where informal coercion might be experienced. However, these items only address severe occurrences of informal coercion. Tools that also explore less severe forms of informal coercion in different situations should be designed to better understand the ‘continuum of coercion’. A psychometrically rigorous French-language tool to measure perceived lived experiences of treatment pressures was recently designed, developed and validated in collaboration with users (Pressures in Psychiatry Scale or P-PSY35) (Golay et al., 2024). The negative consequences of the coercion exerted by professionals or relatives and the need for specific interventions must be prioritised. We hope that studying patients’ perceptions of informal coercion will be a useful step towards implementing and evaluating programmes aimed at reducing any negative consequences of that coercion. More generally, mental health professionals should be encouraged to address this topic and its implications with their patients. Additional guidelines would be especially welcome to protect patients from coercion because their perceptions of not being involved in fair decision-making processes can reinforce their feelings of being coerced, with well-documented detrimental effects (Golay et al., 2024).

Future studies using Rasch analysis would also enable an examination of relationships between the latent continuum of patients’ perceived amounts of coercion experienced and other important sociodemographic and clinical variables.

Despite an adequately large sample and an innovative statistical approach able to consider asymmetric relationships, this study had some limitations. First, the ‘severity’ of coercion was defined as based on its pattern of use and not on patients’ personal views of different types of coercion. Second, informal coercion was assessed somewhat basically, only addressing severe occurrences of informal coercion. Additionally, the statistical implication approach required variables to be binary, and the precise timing and order of occurrences of coercion were not considered. The general precedence of one coercive measure compared to another could nevertheless be estimated. Third, the Rasch analysis required that observed items should be conditionally independent of each other, which is difficult to assess with certainty. The assumptions of local stochastic independence and the assumption of a single underlying dimension may, therefore, be a limitation of Rasch analysis. However, the Iota index does not rely on those assumptions. Fourth, implication was defined as ‘statistical implication’ in the sense that counter-examples were rare. This is distinct from ‘perfect implication’, where counter-examples do not exist. Additionally, statistical implication should be distinguished from causality because implication may be embedded in a complex network of dependencies (Noël, 2021). Fifth, given the self-reported nature of psychiatric diagnoses and information on patients’ history of coercion, our assessments may be imprecise.

## Conclusions

The extent of the use of severe informal coercion may constitute a counter-example to the idea that there is a ‘continuum of coercion’ where formal coercion is implicitly considered the most severe form. In the present study, coercion involving child custody implied that all the other forms of coercion had been considered. Thus, the supposed degree of severity of coercive measures in psychiatry, from informal to formal coercion, may not correspond to the lived experiences of the persons concerned.
